# Artificial intelligence assisted compositional analyses of human abdominal aortic aneurysms *ex vivo*

**DOI:** 10.3389/fphys.2022.840965

**Published:** 2022-08-22

**Authors:** Bjarne Thorsted, Lisette Bjerregaard, Pia S. Jensen, Lars M. Rasmussen, Jes S. Lindholt, Maria Bloksgaard

**Affiliations:** ^1^ Department of Cardiothoracic and Vascular Surgery, Odense University Hospital, Odense, Denmark; ^2^ Department of Clinical Biochemistry and Pharmacology, Odense University Hospital, Odense, Denmark; ^3^ Odense Artery Biobank, Odense University Hospital, Odense, Denmark; ^4^ Center for Individualized Medicine in Arterial Diseases, Odense University Hospital, Odense, Denmark; ^5^ Medical Molecular Pharmacology Laboratory, Cardiovascular and Renal Research Unit, Department of Molecular Medicine, University of Southern Denmark, Odense, Denmark

**Keywords:** abdominal aortic aneurysm, neural network, automated histological image analysis, machine learning, cell detection and segmentation, extracellular matrix

## Abstract

Quantification of histological information from excised human abdominal aortic aneurysm (AAA) specimens may provide essential information on the degree of infiltration of inflammatory cells in different regions of the AAA. Such information will support mechanistic insight in AAA pathology and can be linked to clinical measures for further development of AAA treatment regimens. We hypothesize that artificial intelligence can support high throughput analyses of histological sections of excised human AAA. We present an analysis framework based on supervised machine learning. We used TensorFlow and QuPath to determine the overall architecture of the AAA: thrombus, arterial wall, and adventitial loose connective tissue. Within the wall and adventitial zones, the content of collagen, elastin, and specific inflammatory cells was quantified. A deep neural network (DNN) was trained on manually annotated, Weigert stained, tissue sections (14 patients) and validated on images from two other patients. Finally, we applied the method on 95 new patient samples. The DNN was able to segment the sections according to the overall wall architecture with Jaccard coefficients after 65 epocs of 92% for the training and 88% for the validation data set, respectively. Precision and recall both reached 92%. The zone areas were highly variable between patients, as were the outputs on total cell count and elastin/collagen fiber content. The number of specific cells or stained area per zone was deterministically determined. However, combining the masks based on the Weigert stainings, with images of immunostained serial sections requires addition of landmark recognition to the analysis path. The combination of digital pathology, the DNN we developed, and landmark registration will provide a strong tool for future analyses of the histology of excised human AAA. In combination with biomechanical testing and microstructurally motivated mathematical models of AAA remodeling, the method has the potential to be a strong tool to provide mechanistic insight in the disease. In combination with each patients’ demographic and clinical profile, the method can be an interesting tool to in supportof a better treatment regime for the patients.

## 1 Introduction

Understanding the processes in the vascular wall that eventually lead to abdominal aortic aneurysm (AAA) formation and rupture and thereby a high risk of dying is critical to develop preventive medicine. Rupture of the AAA carries a 65–85% risk of death (Sakalihasan et al., 2018). So far, a medical cure to limit the expansion of the wall has not been found and surgical intervention is the only treatment—a procedure that carries a mortality risk of up to 5% within 30 days postoperatively (Hallin et al., 2001).

Despite extensive research to understand the events leading to AAA formation, the precise pathological mechanism behind the AAA development and expansion is still not fully elucidated. Animal models and histological studies on excised human specimens have provided evidence that an intense inflammatory response with adventitial and medial inflammatory cell infiltration, extracellular matrix remodeling, and smooth muscle cell apoptosis are hallmarks of AAA development ((Quintana and Taylor, 2019), (Dale et al., 2015), (Eliason et al., 2005), (Li et al., 2018; Golledge, 2019)). The inflammatory cell infiltration is characterized by lymphocytes, macrophages, and neutrophils ((Dale et al., 2015), (Li et al., 2018)). Neutrophil infiltration has been shown to occur early in animal AAA models ((Quintana and Taylor, 2019), (Li et al., 2018)) and neutrophil depletion was demonstrated to inhibit AAA formation in mice (Eliason et al., 2005). In human AAA patients, a negative correlation was found between neutrophil catalase activity and aortic size (Ramos-Mozo et al., 2011). Extracellular matrix degradation products and numerous chemokines recruit macrophages to the injury sites (Dale et al., 2015). Recently however, T cells rather than macrophages, where found to be the major leucocyte population in human late-stage AAA with greatest accumulation in the perivascular tissue (PVT) (Sagan et al., 2019). T cells in the pathological damaged AAA wall were in a more activated state (particularly CD 4^+^ cells (T-helper cells)) or dysregulated (especially CD 8^+^ cells) and T cell infiltration in PVT was strongly related to AAA size. A direct role for cytotoxic CD8^+^ T cells in the pathogenesis of AAA has also been proposed in a study were IFN-ɣ-producing CD8^+^ T cells promoted development of aneurysm by enhancing matrix metalloprotease activity and cellular apoptosis in a mouse model of elastase-induced AAA. ((Zhou et al., 2013)).

Thus, histological and immunohistological compositional information may provide essential information to understand the pathological mechanism and in search of medical candidates to limit expansion of human AAAs. However, quantification of histological information from excised human AAA specimens is time consuming. Whole slide histological images of human AAAs are large and manual scoring is cumbersome and well known to introduce bias in scoring ((Aeffner et al., 2017)).

In recent years, automated tissue analysis has been developed to provide more objective and reproducible data in shorter time. Machine learning has been used successfully within pathology to extract relevant information from tumor histology ((Turkki et al., 2019), (Litjens et al., 2016)) such as detection of mitosis ((Veta et al., 2015)) inflammatory cell infiltration in human breast cancer ((Turkki et al., 2016), (Litjens et al., 2016)) and automatic segmentation of epithelium and stroma in colorectal cancer ((Linder et al., 2012), (Litjens et al., 2016)).

Automated tissue image analysis makes it possible to use a consistent and objective set of rules of ie cell classification in whole tissue sections across an entire patient population ((Aeffner et al., 2017)). Further, it overcomes issues with interobserver variability. Machine learning powered image analysis allows extremely accurate classification and segmentation of an image with high through-put compared to human manual quantification (Abels et al., 2019).

Here, we hypothesize that artificial intelligence can support and improve the processing and analysis of histological information of excised human AAA specimens. We present an analysis framework for automatic determination of tissue constituents within sections of excised AAA specimens. Using a deep neural network on QuPath annotated whole image files of histological sections, the extracellular matrix components elastin and collagen as well as relevant immunological cell types such as neutrophils (through immunohistological staining for myeloperoxidase, MPO), cytotoxic T cells (CD8^+^) and macrophages (CD68^+^) is quantified. Since perivascular adipose tissue (PVAT) is receiving increasing attention as an important player in the development of AAA ((Kugo et al., 2019; Sagan et al., 2019), (Kugo et al., 2018), (Folkesson et al., 2017), (Dias-Neto et al., 2018)), we also included detection of perivascular tissue PVT, including PVAT, in our analyses, making it possible to quantify cell types and extracellular matrix components in the wall and PVT compartment, respectively.

## 2 Materials and equipment

### 2.1 Ethics

Human specimens of excised AAA were obtained from patients undergoing elective, open abdominal surgeries for AAA. Patients provided written informed consent. The study was approved by the Regional Committees of Health Research Ethics for Southern Denmark (S-20140202), and experiments were conducted according to the principles expressed in the Declaration of Helsinki of the World Medical Association (World Medical, 2013). Upon excision of the aneurysm biopsy, the tissue was placed in Hank’s Balanced Salt Solution (HBSS; Biological Industries; cat. no. 02-016-1A added 10 mM HEPES (Biological Industries; cat. no.03-025-1B) and kept cold until processed. A small piece of the aneurysm was formalin fixed and paraffin embedded (FFPE).

### 2.2 Isolation and processing of tissues by histology

Five micrometer (5 µm) serial sections of FFPE tissues were stained using standard histological and immunohistological methods at Department of Clinical Pathology, Odense University Hospital (pay per service). Briefly, one tissue section per patient was stained with Weigert’s elastin method (Kiernan, 2015). Following, serial cut sections were immunohistochemically stained using antibodies from Agilent-Dako Denmark. Antigen epitope retrieval (AER) was conducted with Ventana Medical cell conditioning solution CC1 (Ventana^®^, 950-124, purchased through Roche Denmark). Anti-CD8 antibody (A039829-2) was diluted 1:100 and applied following AER. Anti-CD68 antibody (M087601-2) was diluted 1:50 and applied following AER. Anti-MPO antibody (A039829-2) was diluted 1:2000. All stained sections were automatically scanned using a Hamamatsu slide scanner (Hamamatsu Photonics K.K.).

### 2.3 Computer hardware

For annotation we used a standard Windows operating system lab top with QuPath (Bankhead et al., 2017) installed and a Wacom Intuos Pro pen with drawing tablet (Wacom GmbH, Germany). For image analyses we used a stronger computer running with Linux operating system, equipped with 2x Intel Xeon Silver 4214 2.2GHz, 3.2 GHz 12 cores/24 threads, 128 GB (8 × 16 GB) DDR4 RAM, 2.5″ 800 GB SAS Enterprise Class Solid State Drive, and a Nvidia Quadro RTX6000 24 GB Graphics card. The image analysis computer used Python 3.8.2 with our own libraries, AAA_ml (github.com/bjtho08/AAA_ml) and mmciad (github.com/bjtho08/mmciad). In addition, the image analysis computer also had QuPath 0.2.3 and FIJI (Schindelin et al., 2012) installed and was controlled remotely via an SSH terminal and X2Go for graphical work.

### 2.4 Dataset description and input data format

#### 2.4.1 Images for annotation and analyses in QuPath

**FIGURE 1:**
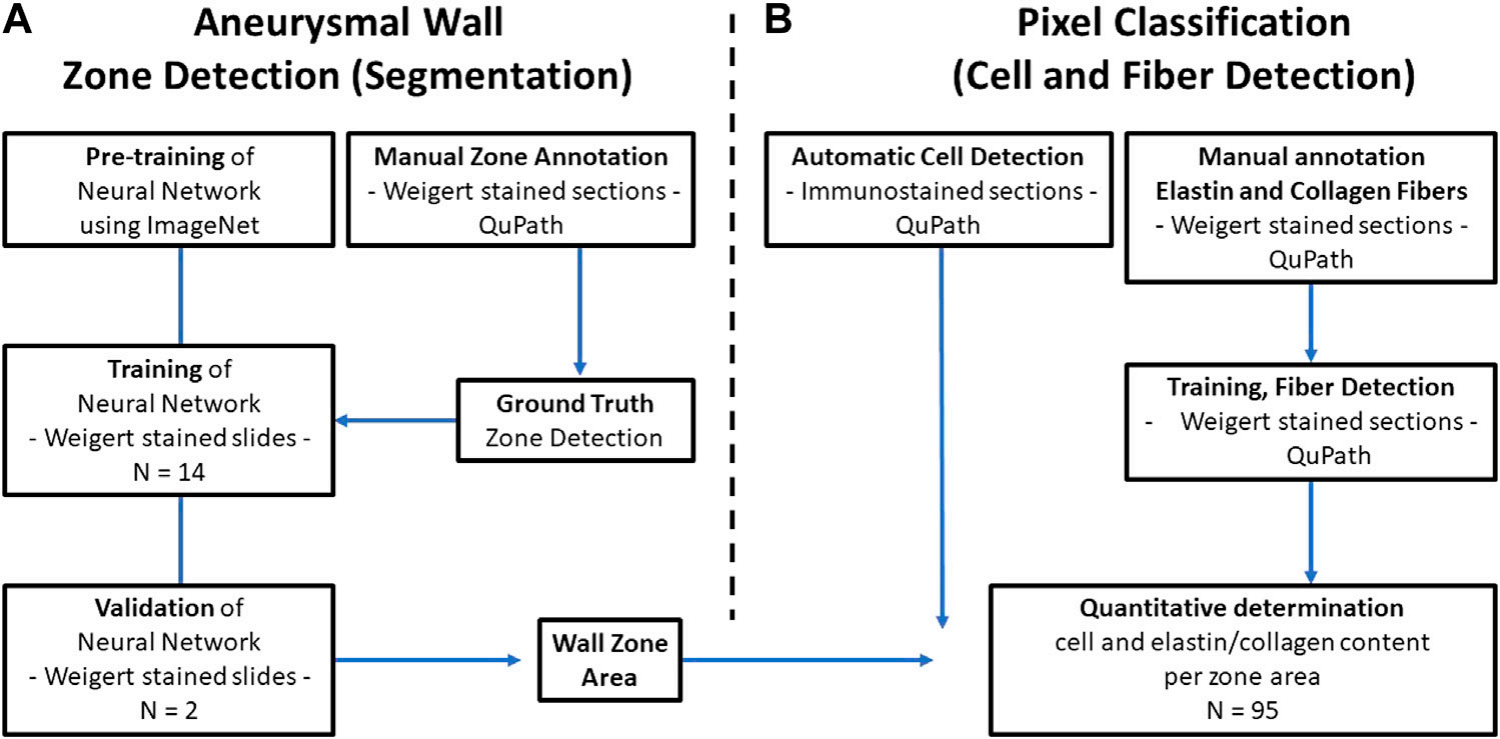
Data analysis flowchart. **(A)** The Deep Neural network is pretrained on ImageNet and trained on basis of manually annotated images of Weigert stained tissue sections of excised AAA specimens from 14 patients. The manual annotation defines the ground truth for the network. The ability of the network to recognize the different defined zones of the AAA wall is validated against manually annotated images from two other patients **(B)** In parallel, QuPath is used for detection of cells and elastin and collagen fibers (right). Finally, by overlaying the defined AAA wall zones and the QuPath output, quantification of the content of specific cells and extracellular matrix fibers per zone is possible.

NanoZoomer Digital Pathology Images (NDPI) were tiled and converted to 24-bit PNG files during the import to QuPath. Our image analysis workflow is outlined in Figure 1. The images were of intact cross sections of the isolated biopsy of the AAA and exhibited large morphological variation. Due to the large degree of histological variation between the patients, and the nature of the electronic data per image as well (2.2 pixels per micron), we considered 16 patient samples adequate for the training and validation. The available patient data was split 14/2 for training/validation purposes.

#### 2.4.2 Images for neural network assisted classification of zones

**FIGURE 2:**
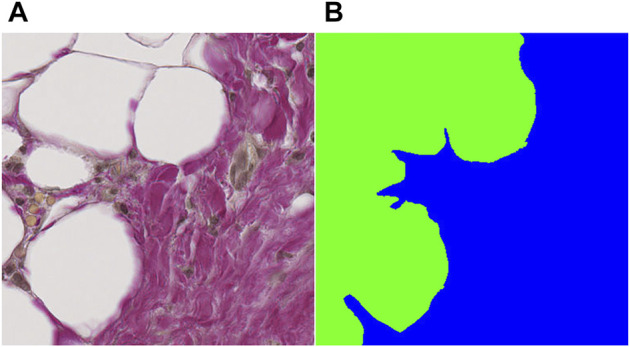
Example of an input tile and a target tile from the mosaic creation process. **(A)** input tile, **(B)** target tile. Both tiles are 384 × 384 pixels (∼450 nm/pixel), which gives an adequate amount of information about the tissue for determining the class label(s) in the field of view.

Each patient sample in the training and validation datasets was converted to a mosaic of overlapping tiles with dimensions 384 × 384 pixels (175 × 175 µm), which were stored as individual PNG images. Tile dimensions must be divisible by the scaling factor S = 2^n^, with n equal to the number of pooling operations, to scale properly throughout the pooling layers. For our network, n = 4, whereby S = 16. The tile dimensions were deliberately chosen such that the physical tile size in µm would be likely to carry enough information about the area in question to enable useful predictions without taking up too much memory. The tile dimensions were similar sizewise to the output dimensions in the original U-net by (Ronneberger et al., 2015). Tile conversion was done as a preparative step prior to loading image tiles in the network. The code pertaining to the network, including the load step is contained in the following code (https://github.com/bjtho08/AAA_ml). This operation was also performed on the corresponding annotations such that the coordinates defining a tile in the original image corresponded to a pair of PNG images. An example of an input tile and a target tile from the mosaic creation process is shown in Figures 2A,B, respectively.

#### 2.4.3 Filtering of tiles in the neural network

Before being written to disk, a filtering algorithm was applied to determine if a tile should be discarded. Filtering was based on the contents of each annotation tile and the “ignore” label was defined because there is a level of uncertainty regarding the borders between different zones. Tiles with more than 90% pixels labeled “ignore” were discarded, whereas tiles with less than 10% pixels labeled “ignore” were included to reduce the problem with class imbalance while minimizing the risk of discarding valuable information from underrepresented labels.

#### 2.4.4 Normalization and augmentation in the neural network

The dataflow from the tiles on disk to the model input layer includes a normalization step. The values used for normalization are the per-channel averages and standard deviations of the entire dataset. For training, there was also an augmentation step (inbuilt in the code for the preparative step), where tile pairs were subjected to one or several transformations, chosen at random from a predetermined list of operations. The transformations were split into two types: those applying geometric transformation and those applying pixel arithmetics. The latter was only applied to the input images, while the former was applied to both input and target. The following arithmetic transformations were available: addition of a random value in the range [−0.07, 0.07] sampled once pr image; multiplication with a random value in the range [0.8, 1.2] sampled once pr image; random pixel dropout, i.e. setting the RGB value to black with a random dropout fraction in the range of [10%, 50%]. The geometric transformations were: up-down flip, left-right flip, affine rotation by a random degree in the range [−90, 90], affine scaling by a random factor in the range [0.8, 1.2], elastic transformation using displacement fields (c.f. (Simard et al., 2003)). For elastic transformation, the sigma parameter was set to 40.0 and alpha was a randomly sampled value in the range [50, 200]. For all the geometric transformations, edge mode was set to “reflect” to preserve continuity. The augmentation step reduces the likelihood of the network overfitting since it is very unlikely to see two identical inputs over the course of the training.

### 2.5 Analysis network

#### 2.5.1 Annotation and simple segmentation in QuPath

Images of the 16 patient samples were manually annotated to generate the ground truth using QuPath (Bankhead et al.) and the Wacom Intuos pro drawing tablet. For definition of different zones in the AAA wall, and for determination of the elastin and collagen area percentages we used the Weigert stained sections. For detection of inflammatory cells, we used the immunostained sections. The finished annotations were exported using a script written in the Apache Groovy language (Supplementary Material S1).

#### 2.5.2 Zone definitions in the AAA wall

**FIGURE 3:**
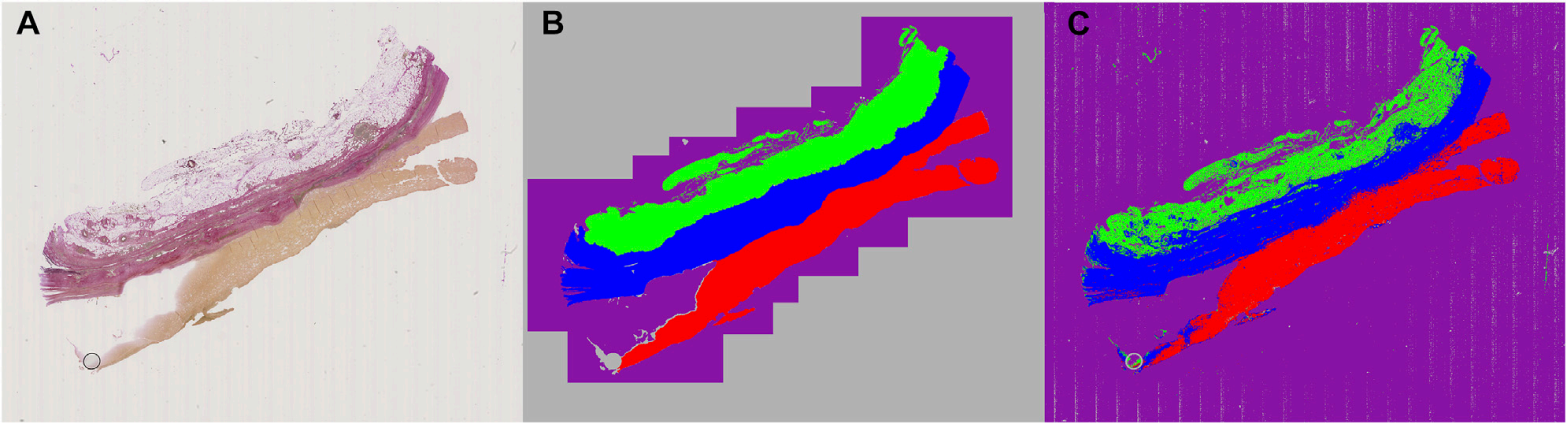
Example of raw, annotated and segmentation prediction image. **(A)** Input image, Weigert stained **(B)** Partially annotated ground truth. The gray area surrounding the purple region is removed from the dataset during training to reduce class imbalance and improve training time **(C)** Segmentation prediction output from the neural model. Color coding: Blue: Zone 1. Green: Zone 2. Red: Thrombus. Purple: Background. Gray: Ignore.

Images of the AAA biopsy were annotated using three classes: “zone 1” for vascular wall tissue, “zone 2” for loose perivascular tissue (PVT) and “thrombus” for thrombus material not cleared at the time of excision during surgery. An example of an input image is shown in Figure 3A, while the partly annotated ground truth is shown in Figure 3B, and the segmentation prediction output from the neural network in Figure 3C. We chose to only annotate part of the background close to the actual sample to make sure the network had enough material to learn from, but without increasing the class imbalance unnecessarily. For simplicity, the surrounding unannotated background was labeled as “ignore”. Several areas within the tissue boundaries were not easily separated into the desired class labels and those were also labeled as “ignore” with the intent of letting the neural network decide where inside these areas of uncertainty the actual borders are located. The annotations were exported as color indexed PNG images.

#### 2.5.3 Neural network assisted classification of zones

**FIGURE 4:**
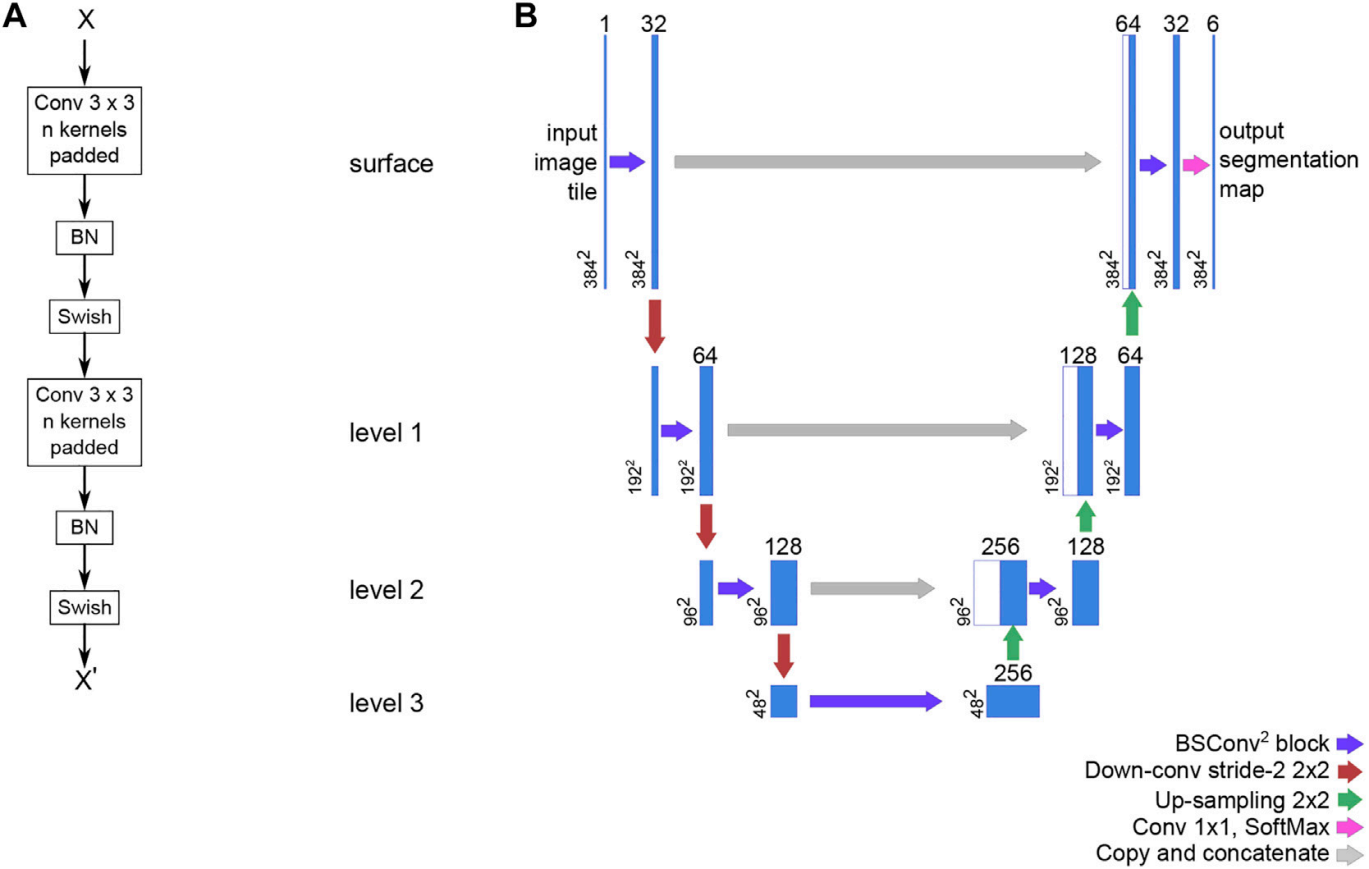
The basic building block of our U-Net variant and the overall network architecture. **(A)** The BSConv2 block consists of two repeats of a reflection padded 3 × 3 convolution and linear activation followed by batch normalization and a trainable Swish activation **(B)** The U-Net architecture is structured into levels that share operation parameters. For a U-net with a depth of three, there are three levels below the surface level (depth 0). For each subsequent level, the number of filters per convolution is doubled and the spatial dimensions are halved.

The neural network is a variation of the U-Net (Ronneberger et al.). Our version is written in TensorFlow 2.1 and uses the Swish trainable activation (Ramachandran et al., 2017) function rather than ReLU, but still pre-trained on the ILSVRC 2012 (ImageNet) dataset (Russakovsky et al., 2015). Additionally, all convolutional layers have been modified to support reflection padding to preserve tensor dimensions throughout the network and avoid discontinuous borders in the data. The code for the network is available as a library through GitHub (https://github.com/bjtho08/mmciad). We used the Talos Hyperparameter Optimization library (Kotila, 2019) to determine the optimal hyperparameters. We used a network depth of 3, and 32 as the base number of filters per convolution. The depth indicates the number of levels below “surface” in the network, while the base number of filters indicate the number of filters per convolution operation in the surface level. The number of filters in the subsequent levels below surface is always twice the number of filters in the level above, meaning that level three will have 256 filters per convolution. We also used batch normalization before activation on each convolution and opted for strided de-/convolutional up- and down-scaling rather than up-sampling and pooling layers. The network model architecture, including the BSConv^2^ building block, is illustrated in Figure 4. The (untrained) network parameters were initialized using the He normal function (He et al., 2015) and then trained using the Adam optimization function (Kingma and Ba, 2014) and Squared Jaccard’s distance (Levandowsky and Winter 1971) as the loss function. Training progress was monitored using the Jaccard similarity index (Jaccard, 1912) and the F_1_ score. These metrics were calculated for both the training and validation datasets. Once the training was completed, the model was stored on disk with the best performing set of parameter weights from the training stage. Following this, the model was evaluated on the validation dataset to generate data for visual quality control and statistical data.

#### 2.5.4 Application of zone classifications in QuPath for further analyses

The output segmentation maps from the neural network were converted to QuPath regions (masks) using a script (Supplementary Material S2) in FIJI (Schindelin et al.) to generate binary maps for each category and reading those back into QuPath using another script (Supplementary Material S3). We created separate QuPath projects for each of the performed stainings to better keep track of which analyses to perform for each staining. The latter script needs to be run once for each QuPath project before any subsequent analyses can take place.

#### 2.5.5 Detection of CD8 and MPO positive cells/zone

QuPath offers a selection of features and intuitive tools to easily annotate whole slide images and perform different analytical operations on the data. These operations include positive cell detection, stain separation/color deconvolution, and pixel classification. Table 1 Shows the relevant settings for performing positive cell detection for CD8 and MPO stained slides, respectively. QuPath will only perform cell detection inside the selected annotation(s), but a small script (Supplementary Materials S4, 5 for CD8 and MPO, respectively) ensures that cells/area or fibers/area are counted in each of zone 1 and 2.

**TABLE 1:** QuPath Positive cell detection parameters for MPO (neutrophils) and CD8 (T-cells) positive detection, respectively. Non-standard values in QuPath are listed, remaining parameters were set to “default” in QuPath.

Parameter	Value	
	MPO	CD8
detectionImageBrightfield	“Hematoxylin OD”	“Hematoxylin OD”
backgroundRadiusMicrons	8.0	10.0
thresholdCompartment	Cytoplasm: DAB OD mean	“Cell: DAB OD mean”
thresholdPositive1	0.2	0.1

#### 2.5.6 Detection of CD68 positive staining/zone

CD68 is detected as an area using a combination of color deconvolution and pixel classification to register all pixels with a DAB optical density value above a certain threshold (Supplementary Material S6).

#### 2.5.7 Determination of the elastin/collagen area percentages in zones one and two

For elastin and collagen fibers, a slightly more advanced pixel classifier is used to recognize the fibers of a specific color and report the total area of a given fiber within each zone. These values are then used to calculate the per zone elastin and collagen area percentages, respectively (Supplementary Material S7).

### 2.6 Application of our analysis network for screening of patient samples

Following validation of our analysis network, we used the model to analyze a total of 95 patient samples different from the ones used for training and validation. Images of Weigert stained sections of each of the 95 patient samples were fed into the trained, validated network and data were retrieved on zone classifications. Zone classifications were applied on images of CD8, CD68 and MPO immunostained sections and cells/zone area was determined for CD8 and MPO immunostainings and stained area/zone area for CD68 immunostainings. Finally, the elastin and collagen area percentages per zone area was retrieved from the Weigert stained sections.

### 2.7 Output data format

Results from each image’s analysis is saved as . csv file format, one file per patient. Data, e.g., the number of CD8 positive cells or the area of CD68 stained tissue are retrieved per zone area.

## 3 Results

### 3.1 Results from the training and validation

We developed a machine learning assisted analysis of histological specimens from curative surgeries of abdominal aortic aneurysms in humans. Excised aneurysmal tissue was formalin fixed and embedded in paraffin, and serial sections were cut. The first of these was stained with Weigert histological stain, the following, adjacent sections with different antibodies, including CD8, CD68, MPO. We used manual annotations on the Weigert stained sections to define three classes: “zone 1” for vascular wall tissue, “zone 2” for loose perivascular tissue (PVT) and “thrombus” for thrombus material not cleared at the time of excision during surgery. The annotated images were fed into a neural network, pre-trained on the ILSVRC 2012 (ImageNet) dataset (Russakovsky et al., 2015). The images of the original staining, partially annotated images and the segmentation prediction output from the neural network are shown in Figures 3A–C, respectively. Images from 14 patients were used for training the network, and images from two different patients were used for validation. Finally, we tested our model on 95 new patient samples to confirm the applicability of the method and show the variation in the output data.

After running a tile-based filtration, the total number of image tiles for training and validation was 76203 and 12636, respectively, giving a training:validation split ratio of 14/2. Thus, the filtration did not alter the training/validation ratio aimed for. Filtering was based on the contents of each annotation tile and was applied to avoid class imbalances. Therefore, several tiles contained small quantities of pixels labeled “ignore” and that is the reason for the presence of “ignore” in the confusion matrix. We tried to convey this by manually adjusting the pixel counts for the affected labels. The results of these adjustments are listed in the bottom two rows of Tables 2, 3, which also show significantly improved performance for the model compared to the unadjusted values.

**TABLE 2:** Classification report for the performance of the network model on the validation dataset. The macro averages are the unweighted means of each metric, while the weighted averages are the support-weighted means. The bottom two rows were calculated with the assumption that all data labeled Ignore are irrelevant to the performance and thus excluded.

Class	Precision	Recall	F_1_-score	Jaccard index	Support
Ignore	0.091	0.070	0.079	0.041	41672692
Zone 1	0.766	0.903	0.829	0.708	265953620
Zone 2	0.858	0.796	0.826	0.704	306283491
Thrombus	0.969	0.887	0.926	0.862	145997791
Background	0.312	0.984	0.474	0.310	760249363
macro avg	0.599	0.728	0.627	0.456	1520156957
weighted avg	0.558	0.897	0.639	0.470	1520156957
excluding ignore					
macro avg	0.726	0.892	0.764	0.618	1478484265
weighted avg	0.572	0.921	0.655	0.487	1478484265

**TABLE 3:** Corrected classification report for the performance of the network model on the validation dataset. The Background (BG) precision has been adjusted to a reasonable estimate to take into account the number of tiles in the input image tile data set without information. The macro averages are the unweighted means of each metric, while the weighted averages are the support-weighted means. The bottom two rows were calculated with the assumption that all data labeled Ignore are irrelevant to the performance and thus excluded. Please see discussion for details.

Class	Precision	Recall	F_1_-score	Jaccard index	Support
Ignore	0.091	0.070	0.079	0.041	41672692
Zone 1	0.766	0.903	0.829	0.708	265953620
Zone 2	0.858	0.796	0.826	0.704	306283491
Thrombus	0.969	0.887	0.926	0.862	145997791
Estimated BG	0.980	0.984	0.982	0.965	760249363
Macro avg	0.733	0.728	0.728	0.573	1520156957
Weighted avg	0.893	0.897	0.894	0.808	1520156957
Excluding ignore					
Macro avg	0.893	0.892	0.891	0.803	1478484265
Weighted avg	0.915	0.921	0.917	0.846	1478484265

**FIGURE 5:**
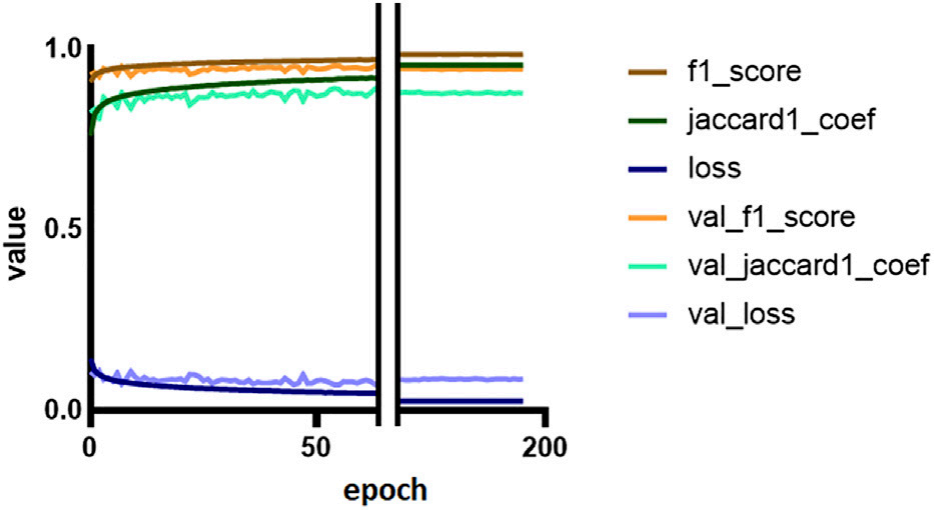
Training and validation metrics per epoch. The Jaccard similarity index (JSI) calculated during training and validation is reduced because of a misconfiguration (see Discussion for details). The F1 and Jaccard scores for training are overall mean measures of the models’ precision and recall. Loss is a measure of costs of the model, and represents how poor the model is.

**FIGURE 6:**
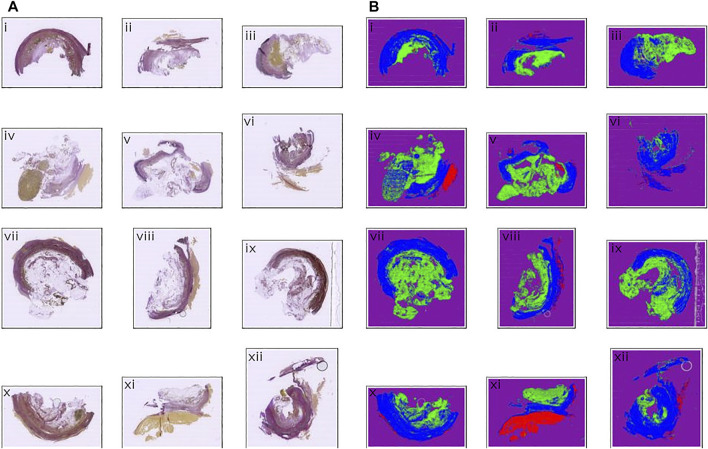
Input and output comparison. **(A)** Input images, Weigert stained **(B)** Segmentation prediction output from the neural model. Color coding: Blue: Zone 1. Green: Zone 2. Red: Thrombus. Purple: Background. Gray: Ignore.

**FIGURE 7:**
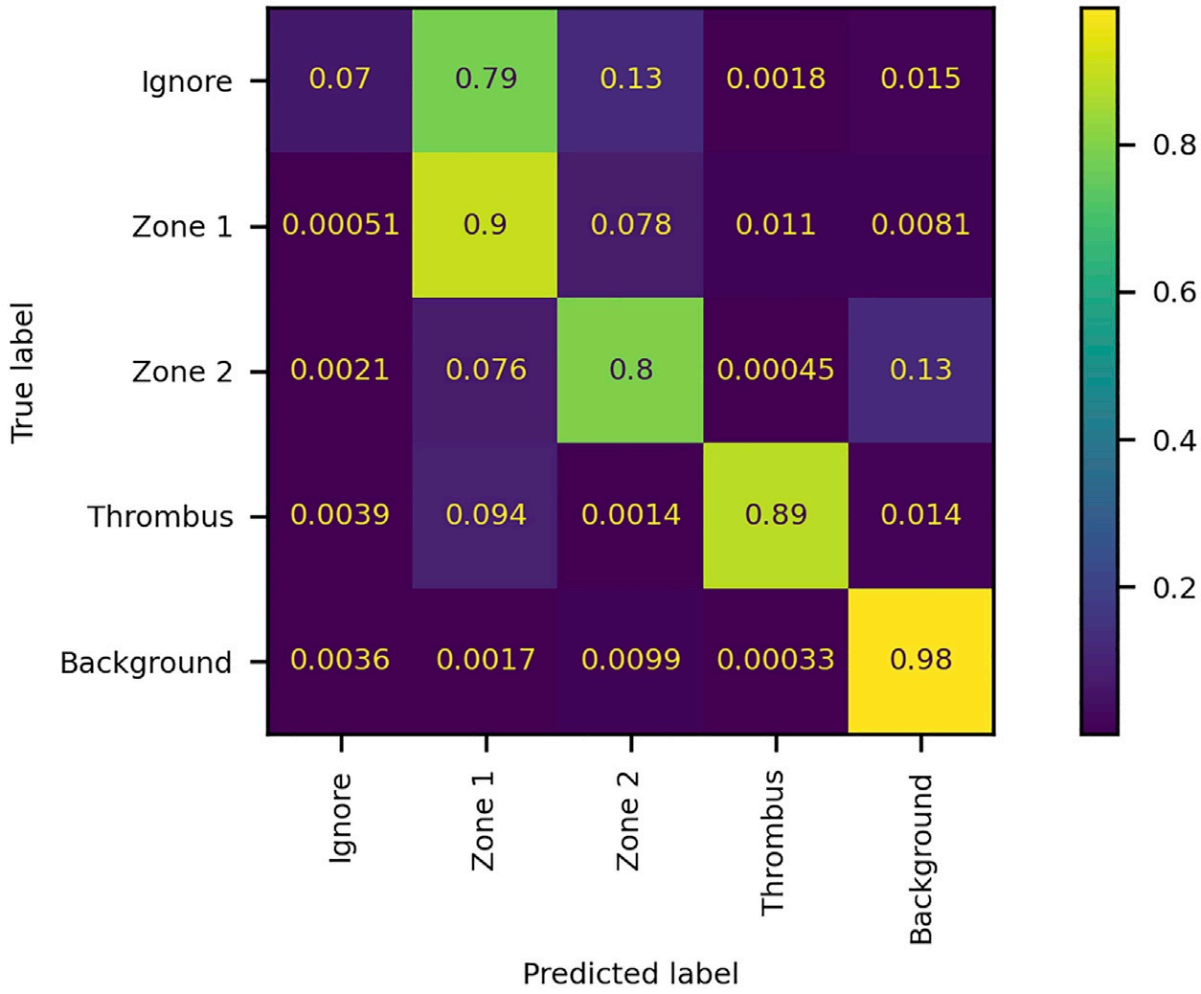
Confusion matrix of the performance of the network model on the validation dataset. The numbers denote the fraction of pixels classified as label x and belonging to label y. The matrix is normalized by true label, i.e., each row summates to 1. The diagonal elements represent true positives, while off-diagonal elements represent false positives (columns) and false negatives (rows).

The model was trained for a total of 200 epochs (not including pretraining) and we used the Talos Hyperparameter Optimization library (Kotila, 2019) to determine the optimal hyperparameters used. These included the number of filters, depth of the network and several loss functions as well. We chose to run the model for 200 epochs to make sure it converges before selecting the epoch with the best performance. Fine-tuning was done by running the model through different number of epochs, and then following each run, evaluating loss and accuracy, aiming at the run with lowest loss and highest accuracy. The model saved after 33 epochs showed the best performance on the validation data set. The evolution of all relevant metrics during training is shown in Figure 5 and the final validation metrics are detailed in Tables 2, 3. Adjusting for background regions and regions classified as ignore (in the ground truths), the weighted average Jaccard similarity index is found to be 84.6%. While Figure 3 gives a brief overview, Figure 6 shows a more detailed example of what the model was trained on (Figure 6A, detailed output segmentation map Figure 6B). By visual inspection, there are a few artifacts in the background and small islands of blue inside the green and red regions, but overall, there appears to be a large overlap between ground truth and prediction. The confusion matrix in Figure 7 supports this visual, qualitative assessment, even though it is based on the uncorrected numbers, not considering that we “discarded” all image tiles without any tissue information in the raw images by assigning them the “Ignore” class (grey in Figure 3B). Barring the “ignore” label, the model is certain of all labels, with at least 80% correct predictions for each. This is also evident from Table 3, where the precision column corresponds to the confusion matrix diagonal, where the corrected numbers give a significantly higher overall score.

### 3.2 Zone classification in 95 patients

**FIGURE 8:**
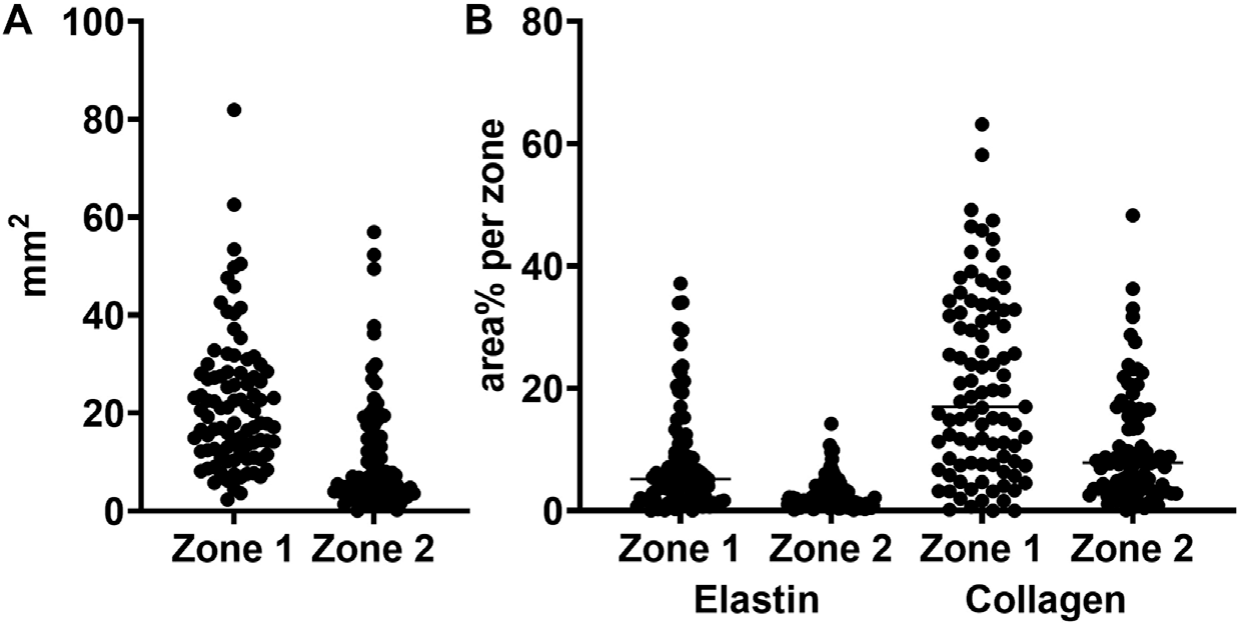
Total area and elastin and collagen area percentages in zones 1 and 2. **(A)** zone area for zone 1 (vascular wall) and zone 2 (perivascular loose connective tissue) **(B)** area percentages for elastin and collagen in zone 1 and 2, respectively.

To evaluate the feasibility of our new method, we applied it to materials from 95 patients different from the 16 used for training and validation. Images of Weigert stained sections were analyzed using the network, and zone1, zone 2 and thrombus defined. Figure 8A shows the distribution of zone 1 and zone 2 areas across the different patients. It is evident that the zone areas are not normal distributed, samples are highly different, as expected.

### 3.3 Extracellular matrix detection

From the Weigert stained tissue sections we determined the elastin and collagen area percentages per zone class. The elastin and collagen detections were performed using a slightly more advanced pixel classifier compared to the immunostainings. The classifier requires either/or, and therefore a decision is taken on whether a pixel contains either elastin or collagen or none of the two. Figure 8B shows the raw area% measurements of the two components of the extracellular matrix per zone 1 and zone 2 for the 95 patients. As above, it is evident that also the content of extracellular matrix, here defined broadly as elastin and collagen, is markedly different between patients.

### 3.4 Cell detection in 95 patients

To allow quantification of the number of immunostained cells per zone and the area of immunostained tissue per zone, the zone classifications defined from the Weigert stainings were applied to images of the immunostained sections. To ensure complete adherence between sequentially sectioned tissues, we first manually visually evaluated the suitability of each mask defined from the Weigert stained sections on the immunostained sections. We assessed the quality of the zone classification since there can be significant discrepancies between location and orientation of the tissues on the histological slides. The complex nature of the imported regions makes it prohibitively memory-intensive to adjust the regions in the cases where the overlap is unsatisfactory.

**FIGURE 9:**
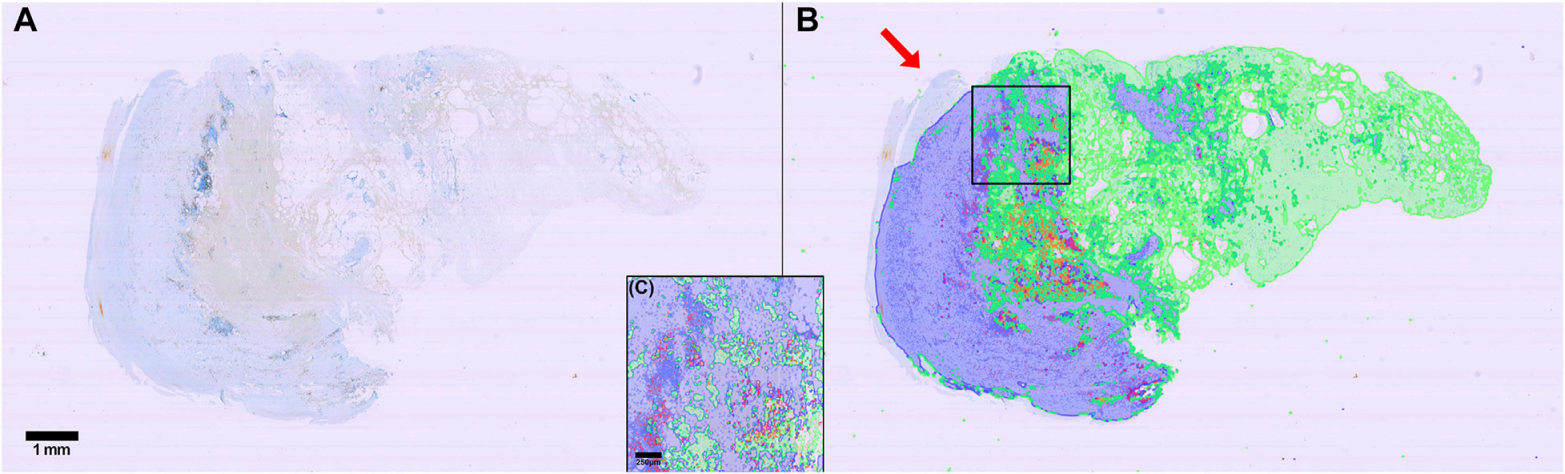
Illustration of cell detection using antiCD8 immunostaining. **(A)** Raw immunostained image (DAB and hematoxylin stained). **(B)** Image in A with overlaid zone predictions (Blue: Zone 1 border. Green: Zone 2 border. Red: Thrombus) and all cell detections (Light blue: Hematoxylin stained nucleus in CD8 negative cell. Light red: Hematoxylin-stained nucleus in CD8 positive cell). **(C)** zoom in of square in **(B)**. Scalebar is 1 mm **(A, B)** 250 µm **(C)** Arrow: part of the tissue that we can assume would be zone 1. Please refer to the discussion for details.

For CD8 positive cell detection, the zone classification segmentation maps were imported using a FIJI script written in ImageJ macro language (Supplementary Material S2) and a QuPath script written in Groovy (Supplementary Material S3). Out of 95 slides, three did not have a corresponding Weigert section, seven were too damaged to work on, one had an unknown read error, one was missing tissue completely, one had a different number of tissue sections, eight were too distorted, and 63 were markedly displaced relative to the Weigert section. The degree of displacement varied from a few tens of pixels to a several hundred pixels. Nine slides were adequately similar to their corresponding Weigert slides to allow application of the zone classification segmentation maps. Cell detection was conducted using Supplementary Material S4 and representative results from one patient is shown in Table 4 for illustration. The results show that most cells are located in zone 1, but a significant portion is found in what is labeled as background. As illustrated in Figure 9 arrow, part of the tissue that we can assume would be zone 1 is outside the zone perimeter and therefore the detected cells in this region are counted towards the background label. The results also show that around 9% of all detected cells are CD8-positive. Zone 2, a region spanning 22 mm^2^ and which consists mostly of adipose tissue, contained only 50 CD8 positive cells.

**TABLE 4:** CD8 positive cell detection for a patient sample. The detections are the total cell count for the indicated class. Detections: number of cells detected per zone class. Positive: number of CD8 positive cells. Positive%: percentage og all detected cells that are CD8 positive. Positive per mm2: number of positive cells per unit area. Area: total area of each class (zone, thrombus or background area). Quite a few cells are detected in “Background”. Please see discussion for details.

Class	Detections	Positive	Positive %	Positive per mm^2^	Area [mm^2^]
Zone 1	129824	12308	9.5	545	22.6
Zone 2	48472	1680	3.5	76	22.0
Thrombus	1920	148	7.7	363	0.4
Background	80	0	0	0	85.0

Similar results with respect to the difficulty of applying the zone classification segmentation maps were observed for the CD68 and MPO immunostained sections, respectively (results not shown).

### 3.5 Time and resources required

The project required purchase of a dedicated image analysis computer for handling the digital images from pathology. Each image is 4.2 GB of data. Annotations can be conducted on a normal personal labtop with QuPath installed. Full manual annotation of one image, for example for zone classification, took 3–5 days full time by an expert. Pretraining of the Network on the ImageNet dataset (Russakovsky et al., 2015) to 3 weeks, while training on the annotated patient samples took a little more than 1 week. Validation of the training took only an hour. Analysis of the 95 patient samples took 1 day plus data extraction and calculations.

## 4 Discussion

We present here an analysis framework in digital pathology for quantification of the content of different inflammatory cells and extracellular matrix constituents in tissue sections of excised human AAA. We applied a DNN for recognition and definition of different zones in the AAA wall, thrombus, vascular wall and perivascular loose tissue. Zone classification segmentation maps were defined using Weigert stained tissue sections and applied on serial immunostained sections. This, however, proved extremely difficult due to marked differences between the serial cut sections: Position of the immunostained sections on the glass slides was markedly different compared to the Weigert stained section, some tissues were rotated, folded, distorted, or had major parts missing, likely due to calcifications making the manual sectioning difficult. Thus, before our method can be applied on a broader analysis scale for quantification of the presence of specific inflammatory cells in specific zones in the AAA wall, we suggest addition of landmark recognition for positioning the zone classification maps. The application of automatic segmentation on the immunostainings however proves highly efficient for quantification of total cell content in intact tissue sections. This is in agreement with previous reports in application of digital pathology on e.g. tissue sections from cancerous tissues (Mi et al., 2020).

Manual quantitative assessment of histological samples is tedious and very resource demanding (manpower and time). Automatic quantification is at present clinically only implemented as cell counts in full blood and bone marrow analyses. However, quantification of tissue components and infiltrative cells in tissues opens a door into better and faster assessments of infiltrative, degenerative, and healing processes of importance for stratification of disease, prognosis and response of medical treatment. Prior studies on a limited number of patient samples have demonstrated the connection between the microstructure and biomechanical properties of the AAA wall (Niestrawska et al., 2019). As we describe above, complete manual annotation of one image took 3–5 days, while we with the final model could run cell detection, for three different stainings, for 95 individual patient samples, in less than a day.

With this method paper, we have made available all scripts and the network code needed for other users to adapt the method to their own needs. We hope, that our computational method in combination with already established methods on *ex vivo* biomechanical testing of fresh, excised AAA specimens and microstructurally informed mathematical models of the wall behavior (e.g. (Bersi et al., 2019; Niestrawska et al., 2019)) can support the pressing need for additional mechanical and microstructural data to better inform material remodeling of the AAA wall. High throughput quantification of histological data are required to develop high-resolution local correlations among multiple mechanical metrics and wall microstructure. Applying the analysis flow in combination with methods in animal models will allow longitudinal studies, c. f. e.g. (Bersi et al., 2019). And although findings from animal models of AAAs cannot be directly translated to human AAAs, animal models of AAA will provide knowledge for further investigation and insight into human AAA disease (Lysgaard Poulsen et al., 2016), eventually, to the benefit of the patients. However, before other users adapt our method, discussion of a few pros and cons is warranted.

### 4.1 Precision of the network (zone recognition)

From the training and validation, we show uncorrected and corrected measures of precision of our network. While the uncorrected results listed in Table 2 reveal a weighted average Jaccard score of only 49%, it is important to note that this is due to a technical shortcoming of the way our data is structured and how the score is calculated. For training purposes, the whole slide images are divided into smaller tiles and filtering was determined based on the contents of the annotation tile. This was done to reduce the problem with class imbalance while minimizing the risk of discarding valuable information from underrepresented labels. Furthermore, this approach allowed the model to decide for itself which label was more appropriate for each of the uncertain pixels at the cost of getting a lower accuracy, because some pixels would appear mislabeled when the model did not label them “ignore”. We tried to convey this by manually adjusting the pixel counts for the affected labels.

The consequence of this choice is that the whole slide image annotation has a large area of actual background (empty image tile/tile with no tissue) incorrectly labeled as “ignore”. During post-training validation, the entire slide is analyzed and the incorrectly labeled “background” is included in the data used to generate the results. Therefore, the precision of the “background” label is dismally low, even though the recall is very high. The same issue is present for the “ignore” label, but here we see both a low precision and a low recall. This is because the background gets predicted correctly by the network, leading the validation to the incorrect conclusion that the recall is quite low. But since the network also learned how to figure out where the edge case pixels belong, the precision is equally low. We tried to account for this in two ways. First, we recalculated the averages by excluding the “ignore” label. Then, we estimated the precision for the “background” label based on the overview shown in Figure 6. The conservative estimate here is that the model gets the background right in at least 98% of the cases. This correction raises the weighted average Jaccard score from the original 0,31 to 0.85. Previous studies (Zou et al., 2004; Gu and Li, 2021) have reported that for biomedical image segmentation, a DICE score of 0,7 or higher is sufficient. DICE scores can be converted to Jaccard scores using formula J = D/(2-D), where D is the DICE score, and J is the Jaccard score. Thus, we need to surpass 0.7/(2-0.7) = 0.538 in our Jaccard index, which we are well above.

### 4.2 Application of zone class segmentation maps to serial, immunostained sections

The segmentation maps generated by our model for the 95 patient samples were converted to QuPath regions imported into the corresponding CD8 patient samples. To get an overview, we first performed a qualitative assessment of the overlap between the segmentation maps and the tissue showed that out of 95 patient samples, only nine had an adequate overlap. This was visible by the human eye. The assessment was based on both location, rotation, macroscopic morphological similarity, and quality of the tissue in both the Weigert stainings and the CD8 immunostainings. With a success rate of roughly only 10% the usability of the zone class segmentation maps in the immunostained serial sections was highly disappointing. This is a major drawback to our method. Furthermore, manual inspection of the suitability of each zone classification segmentation map retains the histological analyses susceptible to personal bias. We aim at having a digital validation of our visual evaluation of the zone classification segmentation maps. This, in our opinion, requires two improvements: First, extreme care must be taken already at the point of sectioning of the tissues, to collect the sections and position them neatly and precisely on the glass slides, and discard sections that are folded, or destroyed by the microtome. Second, we suggest adding to our method a module of image recognition using landmark recognition and registration followed by thin-spline transformations to have the computer automatically detect and match the zone classification segmentation maps with the immunostainings. The nature of the AAA tissue sections is however complicated to work with also in this respect, since we observed many sections being torn apart during mechanical sectioning due to calcification deposits in the vascular wall.

### 4.3 False high cell count in “background” label

Due to the difficulty with applying the zone class segmentation maps from the Weigert stainings on the immunostainings, we report a false high number of cells in the background. This can be overcome by applying a module with landmark recognition to increase the overlap between the zone classes and the tissue on which they are applied. For users not in need of dividing the tissue section in zones, for us vascular wall and perivascular loose connective tissue, zone class segmentation will never be an issue and all users can in principle apply the method for detection of imunostained cells and different fibers in the extracellular matrix. We provide no accuracies on the detection of CD8, CD68, MPO and elastin and collagen since detection here is deterministic. Thus, defining a ground truth will be too elaborate, and all users can apply the parameters we describe and retrieve similar segmentation results. Furthermore, functional, and reproducible use of QuPath, even among users of limited experience of digital pathology, has been documented to be more accurate than manual scoring (Loughrey et al., 2018).

### 4.4 Perspectives - applicability to other tissues, species and beyond

An absolute requirement for adapting our method is a dedicated image analysis computer with a specialised graphics card and excellent memory. Annotations can, as we described, be performed on a normal lab top, but analyses need more computer power. We also recommend having a person involved who knows about machine learning, artificial intelligence, and who is highly skilled in coding. This person can team up with the specialists in histology and AAA (patho)physiology and adjust our published materials to adapt our neural network for use on other tissues including materials from experimental animal models. Since the network has been extensively pretrained it should only require four to five images of a new type of histology to train the network to perform the histopathological analyses. Addition of other analysis modules in the framework will not require too much additional work, and since several prior reports have suggested the importance of adipose tissue in the AAA wall (Kugo et al., 2018; Kugo et al., 2019; Niestrawska et al., 2019), (Folkesson et al., 2017; Dias-Neto et al., 2018; Doderer et al., 2018; Sagan et al., 2019) a natural next step will be to implement this in the network.

Machine Learning has been used successfully for quantification of tumor-infiltrating immune cells in H&E stained breast cancer whole slide images (e.g. (Turkki et al., 2016) (Litjens et al., 2016; Mi et al., 2020)). In the present study, detection of the two zones in the Weigert stained sections performed well, as did cell detection QuPath. With other tissues of less fibrous and calcified nature than the human AAA, the zone classification segmentation may prove useful and easy to apply as well.

## 5 Conclusion

We present here an image analysis framework to support high throughput analyses of AAA patient histological specimens excised during surgeries. We confirm our hypothesis that artificial intelligence can support and improve the processing of histological information of excised human AAA specimens. Our method can accurately outline the overall architecture of the human AAA and recognize selected immune response cells and matrix constituents both in the vascular wall and perivascular loose connective tissue. Application of zone classification segmentation maps defined from one tissue section to the next turned out to be extremely difficult and calls upon addition of landmark recognition to our model. Each tissue section contains an enormous amount of information and large numbers of cells of different type (defined by the immunostaining applied) could be completed on basis of image segmentation. This can fulfill our need to obtain statistically and clinically relevant information on each patients’ tissue biopsy. With the complete model we foresee use of our analysis approach in personalized medicine. We provide all scripts, code and information required for other user to add this analysis framework to their existing methods. Hopefully, the quantification of histological sections in combination with biomechanical testing and microstructurally motivated mathematical models of the AAA remodeling will provide clinically relevant and mechanistic insight into the disease and eventually a better treatment regime for the individual patients.

## Data Availability

The image dataset for this work can be shared following reasonable request to the corresponding author and after approval by the authors and the Odense University Hospital GDPR office. Since the raw image files contain patient information, data exchange will be subject to a data processor agreement between the hospital and collaborating partner(s).
