# Supplementary material for: Artificial intelligence assisted compositional analyses of human abdominal aortic aneurysms ex vivo
Source: Front Physiol. 2022 Aug 22;13:840965. doi: 10.3389/fphys.2022.840965 (PMC9441486; doi:10.3389/fphys.2022.840965)
Supplement: Supplementary file 2 [file DataSheet2.PDF]

```
directory = "/nb_projects/AAA_ml/results/2021-10-09/";//getDirectory("Select input directory");
outputDir = "/nb_projects/qupath_Projects/zonepredictions/masks/";//getDirectory("Choose output directory");
filelist = getFileList(directory);
setBatchMode("hide");
for (i = 0; i < lengthOf(filelist); i++) {
	print(i);
	print(filelist[i]);
    if (endsWith(filelist[i], ".png")) {
	open(directory + filelist[i]);
		name = getTitle();
		print(outputDir + name);
		getDimensions(w, h, channels, slices, frames);
		downsample = 1;
		classes = newArray("Ignore", "Zone 1", "Zone 2", "Thrombus", "Background");
		minThres = newArray(1, 2, 3, 4, 5);//newArray(170, 25, 140, 71, 65);
		maxThres = newArray(1, 2, 3, 4, 5);//newArray(180, 30, 150, 75, 70);
		run("Grays");
		//run("Conversions...", "scale weighted");
		//if (bitDepth() == 8) {
		//	run("RGB Color");
		//}
		//run("8-bit Color", "number=256");
		run("Median...", "radius=10");
		//run("8-bit");
		for (c = 0; c < classes.length; c++) {
			selectWindow(name);
			setThreshold(minThres[c], maxThres[c]);
			run("Create Mask");
			saveAs("PNG", outputDir + name + "_" + classes[c] +"_" + "("+ downsample + ",0,0," + w + "," + h + 
")-mask.png");
			maskWindow = getTitle();
			close(maskWindow);
		}
		close(name);
    }
}
setBatchMode("exit and display");
```
