# Supplementary material for: Artificial intelligence assisted compositional analyses of human abdominal aortic aneurysms ex vivo
Source: Front Physiol. 2022 Aug 22;13:840965. doi: 10.3389/fphys.2022.840965 (PMC9441486; doi:10.3389/fphys.2022.840965)
Supplement: Supplementary file 4 [file DataSheet6.PDF]

```
import qupath.lib.gui.tools.MeasurementExporter
import qupath.lib.objects.PathAnnotationObject
import qupath.lib.roi.ShapeSimplifier

// Create shape simplifier
def simplifyer = new ShapeSimplifier()

// Separate each measurement value in the output file with a tab ("\t")
def separator = ";"

// Choose the columns that will be included in the export
// Note: if 'columnsToInclude' is empty, all columns will be included
def columnsToInclude = new String[]{"Image","Name","CD68: CD68-negative %","CD68: CD68-negative area
Âµm^2","CD68: CD68-positive %","CD68: CD68-positive area Âµm^2","Area Âµm^2"}

// Choose the type of objects that the export will process
// Other possibilities include:
//    1. PathAnnotationObject
//    2. PathDetectionObject
//    3. PathRootObject
// Note: import statements should then be modified accordingly
def exportType = PathAnnotationObject.class

def imagesToExport = [getProjectEntry()]
def imageData = getCurrentImageData()
def hierarchy = imageData.getHierarchy()
def annotations = hierarchy.getAnnotationObjects()
float elevation = 10.0

// Choose your *full* output path
def name = GeneralTools.getNameWithoutExtension(imageData.getServer().getMetadata().getName())
def outputPath = "/nb_projects/qupath_Projects/CD68Detection/measurements/" + name + "_CD68_measurements.csv"
def outputFile = new File(outputPath)

hierarchy.getSelectionModel().clearSelection()
for (annotation in annotations) {
   annotation.setROI(ShapeSimplifier.simplifyShape(annotation.getROI(), elevation))
   hierarchy.getSelectionModel().setSelectedObject(annotation)
   runPlugin('qupath.lib.plugins.objects.RefineAnnotationsPlugin', '{"minFragmentSizeMicrons": 120.0,
"maxHoleSizeMicrons": 120.0}')
   print annotation.getPathClass()
   if (["Zone 1", "Zone 2", "Thrombus"].contains(annotation.getPathClass().name) ) {
      addPixelClassifierMeasurements("New CD68 Detector", "CD68")
   }
}
// Save the detections before exporting
getProjectEntry().saveImageData(imageData)

// Create the measurementExporter and start the export
def exporter  = new MeasurementExporter()
                  .imageList(imagesToExport)            // Images from which measurements will be exported
                  .separator(separator)                 // Character that separates values
                  .includeOnlyColumns(columnsToInclude) // Columns are case-sensitive
```

```
        .exportType(exportType)             // Type of objects to export
        .exportMeasurements(outputFile)       // Start the export process

print "Done!"
```
