# Supplementary material for: Artificial intelligence assisted compositional analyses of human abdominal aortic aneurysms ex vivo
Source: Front Physiol. 2022 Aug 22;13:840965. doi: 10.3389/fphys.2022.840965 (PMC9441486; doi:10.3389/fphys.2022.840965)
Supplement: Supplementary file 5 [file Table1.DOCX]

Artificial intelligence assisted compositional analyses of human abdominal aortic aneurysms ex vivo

**Supplementary files**

Bjarne Thorsted^1^, Lisette Bjerregaard^1^, Pia S. Jensen^2,3,4^, Lars M. Rasmussen^2,3,4^, Jes S. Lindholt^1,4^, Maria Bloksgaard^5,*^

^1^Department of Cardiothoracic and Vascular Surgery, Odense University Hospital, J. B. Winsløws Vej 4, 5000 Odense C, Denmark

^2^Department of Clinical Biochemistry and Pharmacology, Odense University Hospital, Sdr. Boulevard 29, 5000 Odense C, Denmark

^3^Odense Artery Biobank, Odense University hospital, Sdr. Boulevard 29, 5000 Odense C, Denmark

^4^Center for Individualized Medicine in Arterial Diseases, Odense University Hospital, Sdr. Boulevard 29, 5000 Odense C, Denmark

^5^Medical Molecular Pharmacology Laboratory, Cardiovascular and Renal Research Unit, Department of Molecular Medicine, University of SOuhthern Denmark, J. B.Winsløws Vej 21,3, 5000 Odense C, Denmark

*** Correspondence:** Maria Bloksgaard, Ph.D. [mbloksgaard@health.sdu.dk](mailto:mbloksgaard@health.sdu.dk)

**Supplementary file 1: script 1.** This script exports the finished annotations.

**Supplementary file 2: script 2.** This script converts the output segmentation maps from the neural network to generate binary maps for each category. The script runs in FIJI.

**Supplementary file 3: script 3.** This script, run in QuPath, re-imports the binary maps for each category.

**Supplementary file 4: script 4.** This script ensures that cells/area are counted in each of zone 1 and 2.

**Supplementary file 5: script 5.** This script ensures that area of extracellular fibers/area are counted in each of zone 1 and 2.

**Supplementary file 6: script 6.** This script is used for CD68 detection. It is used for detection of an area using a combination of color deconvolution and pixel classification to register all pixels with a DAB optical density value above a certain threshold.

**Supplementary file 7: script 7.** This script is used for detection of elastin and collagen fibers. It applies a slightly more advanced pixel classifier to recognize the fibers of a specific color and report the total area of a given fiber within each zone.
