# Supplementary material for: Artificial intelligence assisted compositional analyses of human abdominal aortic aneurysms ex vivo
Source: Front Physiol. 2022 Aug 22;13:840965. doi: 10.3389/fphys.2022.840965 (PMC9441486; doi:10.3389/fphys.2022.840965)
Supplement: Supplementary file 6 [file DataSheet3.PDF]

```
/**
 * Script to import binary masks & create annotations, adding them to the current object hierarchy for QuPath > 0.2*
 *
 * It is assumed that each mask is stored in a PNG file in a project subdirectory called 'masks'.
 * Each file name should be of the form:
 *   [Short original image name]_[Classification name]_([downsample],[x],[y],[width],[height])-mask.png
 *
 * Note: It's assumed that the classification is a simple name without underscores, i.e. not a 'derived' classification
 * (so 'Tumor' is ok, but 'Tumor: Positive' is not)
 *
 * The x, y, width & height values should be in terms of coordinates for the full-resolution image.
 *
 * By default, the image name stored in the mask filename has to match that of the current image - but this check can be
turned off.
 *
 * @author Benjamin Pavie - greatly inspired from Pete Bankhead's script writen for version 0.1*
 */

 //Requirement:
//QuPath version > 0.2.*
//See https://qupath.readthedocs.io/en/latest/docs/scripting/overview.html

import ij.measure.Calibration
import ij.plugin.filter.ThresholdToSelection
import ij.process.ByteProcessor
import ij.process.ImageProcessor
import qupath.lib.objects.PathObjects
import qupath.lib.regions.ImagePlane
import javax.imageio.ImageIO

// Get the main QuPath data structures
// Get the main QuPath data structures
imageData = getCurrentImageData()
hierarchy = imageData.getHierarchy()
server = imageData.getServer()
plane = getCurrentViewer().getImagePlane()

// Only parse files that contain the specified text; set to '' if all files should be included
// (This is used to avoid adding masks intended for a different image)
includeText = server.getMetadata().getName().replaceFirst(" [\\d\\.]{8}\\.ndpi", '')
prefixes = ["AB 62 CD68-PG 1-50 ", "CD8 AB 24 1-100 ", "AB 186 MPO 1-2000 "]
for (prefix in prefixes) {
   includeText = includeText.replaceFirst(prefix, '')
}
print includeText
// Get a list of image files, stopping early if none can be found
pathOutput = QPEx.buildFilePath(QPEx.PROJECT_BASE_DIR, 'masks')
dirOutput = new File(pathOutput)
if (!dirOutput.isDirectory()) {
   print dirOutput + ' is not a valid directory!'
   return
}
files = dirOutput.listFiles({f -> f.isFile() && f.getName().contains(includeText) && f.getName().endsWith('-mask.png')
```

```
} as FileFilter) as List
if (files.isEmpty()) {
    print 'No mask files found in ' + dirOutput
    return
}

// Create annotations for all the files
annotations = []
files.each { file ->
    try {
        annotations << parseAnnotation(file, plane)
    } catch (Exception e) {
        print 'Unable to parse annotation from ' + file.getName() + ': ' + e.getLocalizedMessage()
    }
}

// Add annotations to image
hierarchy.addPathObjects(annotations)

print "Done!"
/**
 * Create a new annotation from a binary image, parsing the classification & region from the file name.
 *
 * Note: this code doesn't bother with error checking or handling potential issues with formatting/blank images.
 * If something is not quite right, it is quite likely to throw an exception.
 *
 * @param file File containing the PNG image mask.  The image name must be formatted as above.
 * @return The PathAnnotationObject created based on the mask & file name contents.
 */
def parseAnnotation(File file, ImagePlane plane) {
    // Read the image
    original = ImageIO.read(file)
    w = original.getWidth()
    h = original.getHeight()
    //def byteImage = new Byte[w * h]
    img = new BufferedImage(w, h, BufferedImage.TYPE_BYTE_GRAY)
    original.copyData(img.getRaster())

    // Split the file name into parts: [Image name, Classification, Region]
    parts = file.getName().replace('-mask.png', '').split('_')

    // Discard all but the last 2 parts - it's possible that the original name contained underscores,
    // so better to work from the end of the list and not the start
    classificationString = parts[-2]
    if (classificationString == "Ignore") {
        classificationString += "*"
    }

    // Extract region, and trim off parentheses (admittedly in a lazy way...)
    regionString = parts[-1].replace('(', '').replace(')', '')

    // Create a classification, if necessary
    pathClass = null
    if (classificationString != 'None') {
```

```
        pathClass = PathClassFactory.getPathClass(classificationString)
    }

    // Parse the x, y coordinates of the region - width & height not really needed
    // (but could potentially be used to estimate the downsample value, if we didn't already have it)
    regionParts = regionString.split(',')
    double downsample = regionParts[0] as double
    int x = regionParts[1] as int
    int y = regionParts[2] as int
    int wSource = regionParts[3] as int
    int hSource = regionParts[4] as int
    if (w != wSource || h != hSource) {
        downsample = wSource/w
    }

    // To create the ROI, travel into ImageJ
    def bp = new ByteProcessor(img)

    bp.setThreshold(127.5, Double.MAX_VALUE, ImageProcessor.NO_LUT_UPDATE)
    def roiIJ = new ThresholdToSelection().convert(bp)

    // Convert ImageJ ROI to a QuPath ROI
    // This assumes we have a single 2D image (no z-stack, time series)
    // Currently, we need to create an ImageJ Calibration object to store the origin
    // (this might be simplified in a later version)
    def cal = new Calibration()
    cal.xOrigin = -x/downsample
    cal.yOrigin = -y/downsample
    def roi =     IJTools.convertToROI(roiIJ,cal, downsample,plane)
    def pathAnnotation = PathObjects.createAnnotationObject(roi)
    pathAnnotation.setPathClass(pathClass)

    // return the object
    return pathAnnotation
}
```
