# Supplementary material for: Artificial intelligence assisted compositional analyses of human abdominal aortic aneurysms ex vivo
Source: Front Physiol. 2022 Aug 22;13:840965. doi: 10.3389/fphys.2022.840965 (PMC9441486; doi:10.3389/fphys.2022.840965)
Supplement: Supplementary file 7 [file DataSheet1.PDF]

```
/**
 * Script to export annotations to indexed PNG images along with the original pixels in TIFF format
 *
 * The file names will have the following format: (original filename)[.ome].(png|tif), where the brackets
 * signify static elements and the square brackets indicate added only when circumstances require
 * (ie. only for OME-TIFF files). The pipe symbol, |, indicates that one of the two options will be used
 * again depending on the circumstances (png for annotations and tif for original pixels).
 *
 *
 * @author Bjarne Thorsted - greatly inspired from Pete Bankhead's script writen for version 0.1*
 */

 //Requirement:
//QuPath version > 0.2.*
//See https://qupath.readthedocs.io/en/latest/docs/scripting/overview.html

import qupath.lib.images.servers.LabeledImageServer

def imageData = getCurrentImageData()
def server = getCurrentServer()

// Define output path (relative to project)
def outputDir = buildFilePath(PROJECT_BASE_DIR, 'export')
mkdirs(outputDir)
def name = GeneralTools.getNameWithoutExtension(imageData.getServer().getMetadata().getName())
def labelPath = buildFilePath(outputDir, name + ".png")
def originalPath = buildFilePath(outputDir, name + ".tif")
def OMEPath = buildFilePath(outputDir, name + ".ome.tif")
def labelOMEPath = buildFilePath(outputDir, name + ".ome.png")

// Define how much to downsample during export (may be required for large images)
double downsample = 1

// Create an ImageServer where the pixels are derived from annotations
def labelServer = new LabeledImageServer.Builder(imageData)
  .backgroundLabel(0, ColorTools.makeRGB(180, 180, 180)) // Specify background label (usually 0 or 255)
  .downsample(downsample)    // Choose server resolution; this should match the resolution at which tiles are exported
  .addLabel('Ignore*', 1)      // Choose output labels (the order matters!)
  .addLabel('Zone 1', 2)
  .addLabel('Zone 2', 3)
  .addLabel('Thrombus', 4)
  .addLabel('Background', 5)
  .multichannelOutput(false)  // If true, each label refers to the channel of a multichannel binary image (required for
multiclass probability)
  .build()

// Write the image
try {
    print "Writing image and annotations as regular TIFF/PNG pair..."
    //throw new IOException("Just make this fail!")
    writeImage(server, originalPath)
    writeImage(labelServer, labelPath)
} catch (IOException e1) {
```

```
    try {
        print e1
        print "Writing cropped image and annotations as OME-TIFF/PNG pairs"
        def regions_of_int = getAnnotationObjects().findAll {it.getPathClass() == null}
        if (regions_of_int.size() != 1) {
            throw new NullPointerException("unclassified ROI missing")}
        for (annotation in regions_of_int) {
            def region = RegionRequest.createInstance(
                labelServer.getPath(), downsample, annotation.getROI())
            writeImageRegion(labelServer, region, labelOMEPath)
            writeImageRegion(server, region, OMEPath)
        }
    } catch (IOException | NullPointerException e2) {
        print e2
        print "Writing tiles  as TIF/PNG pairs..."
        def pathOutput = buildFilePath(outputDir, 'tiles')
        mkdirs(pathOutput)
        // Create an exporter that requests corresponding tiles from the original & labelled image servers
        new TileExporter(imageData)
                .downsample(downsample)     // Define export resolution
                .imageExtension('.tif')     // Define file extension for original pixels (often .tif, .jpg, '.png' or '.ome.tif')
                .tileSize(10400,9200)      // Define size of each tile, in pixels
                .labeledServer(labelServer) // Define the labeled image server to use (i.e. the one we just built)
                .annotatedTilesOnly(true)   // If true, only export tiles if there is a (classified) annotation present
                .overlap(64)                // Define overlap, in pixel units at the export resolution
                .includePartialTiles(false)
                .writeTiles(pathOutput)     // Write tiles to the specified directory
    }
}

print "Done!"
```
